# Vascular Endothelial Growth Factor remains unchanged in cerebrospinal fluid of patients with Alzheimer’s disease and vascular dementia

**DOI:** 10.1186/s13195-018-0385-8

**Published:** 2018-06-23

**Authors:** Ananya Chakraborty, Madhurima Chatterjee, Harry Twaalfhoven, Marta Del Campo Milan, Charlotte E. Teunissen, Philip Scheltens, Ruud D. Fontijn, Wiesje M. van Der Flier, Helga E. de Vries

**Affiliations:** 1 Department of Molecular Cell Biology and Immunology, Amsterdam Neuroscience, VU University Medical Center, De Boelelaan 1108, 1007 MB Amsterdam, The Netherlands; 2 Department of Clinical Chemistry, VU University Medical Center, Amsterdam, The Netherlands; 3 Alzheimer Centre and Department of Neurology, Amsterdam Neuroscience, VU University Medical Center, Amsterdam, The Netherlands; 4 Department of Epidemiology and Biostatistics, Amsterdam Neuroscience, VU University Medical Centrer, Amsterdam, The Netherlands

**Keywords:** Vascular endothelial growth factor, Alzheimer’s disease, Vascular dementia, Biomarker, Cerebrospinal fluid, Cerebral vascular dysfunction

## Abstract

**Background:**

Increasing evidence suggests that cerebral vascular dysfunction is associated with the early stages of Alzheimer’s disease (AD). Vascular endothelial growth factor (VEGF) is one of the key players involved in the development and maintenance of the vasculature. Here, we hypothesized that VEGF levels in cerebrospinal fluid (CSF) may be altered in AD patients with vascular involvement, characterized by the presence of microbleeds (MB), and in vascular dementia (VaD) patients compared to controls.

**Methods:**

VEGF levels were determined by electrochemilumiscence Meso Scale Discovery (MULTI-SPOT Assay System) in CSF from age-matched groups of controls with subjective cognitive decline (*n* = 21), AD without MB (*n* = 25), AD with MB (*n* = 25), and VaD (*n* = 21) patients.

**Results:**

The average level of VEGF in the different groups was 2.8 ± 1 pg/ml CSF. Adjusted for age and gender, no significant differences were detected between groups (*p* > 0.5). However, we detected a significant correlation between the concentration of VEGF in the CSF and age (*r* = 0.22, *p* = 0.03). In addition, males (*n* = 54) revealed higher VEGF levels in their CSF compared to females (*n* = 38) (males = 3.08 ± 0.769 pg/ml (mean ± SD), females = 2.6 ± 0.59; *p* = 0.006), indicating a gender-related regulation.

**Conclusion:**

Our study suggests that VEGF levels in the CSF do not reflect the cerebral vascular alterations in either AD or VaD patients. The observed associations of VEGF with age and gender may indicate that VEGF reflects normal aging and that males and females may differ in their aging process.

**Electronic supplementary material:**

The online version of this article (10.1186/s13195-018-0385-8) contains supplementary material, which is available to authorized users.

## Background

Cerebrovascular disease is a major contributor to cognitive decline and dementia in old age [1], and vascular dysfunction may contribute to early onset of dementia and progression thereof [2]. Vascular damage, including that of the blood–brain barrier (BBB), is believed to be the result of impaired cerebral blood flow (CBF), which in turn induces hypoperfusion of the brain, resulting in hypoxia [2–5]. In addition, alterations in cerebral hemodynamics may impair glucose transport to the brain and reduce cerebral perfusion, propagating the process of neurodegeneration [6–8]. Postmortem reports further suggest the loss of structural integrity of the cerebrovasculature in Alzheimer’s disease (AD) patients compared to their age-matched peers [9, 10]. Additionally, an increased risk of morbidity is reported in AD patients with vascular diseases, such as atherosclerosis and stroke [11]. However to date, diagnostic tools to assess altered vascular function of the CNS in AD are limited.

Neuropathologically, AD is characterized by the presence of neurofibrillary tangles (NFT) and senile plaques, formed by deposits of beta-amyloid (Aβ) in the brain parenchyma [12]. Many patients with VaD also have AD-related pathology, often referred to as mixed pathology. This vascular pathology includes ischemic changes, which if severe enough may also cause vascular dementia (VaD) on their own. In addition, Aβ may accumulate in the walls of cerebral vessels, a process that reflects a direct link between Alzheimer pathology and vessel pathology and is referred to as cerebral amyloid angiopathy (CAA) [13]. CAA may lead to intracerebral hemorrhage, and on MRI microbleeds are often regarded as an indication of underlying CAA [14].

Current body fluid diagnostic biomarkers for AD used in the clinic include the determination of levels of CSF Aβ42, which reflects the presence of parenchymal senile plaque aggregates, in combination with increased levels of total tau (tTau) and phosphorylated tau (pTau) that reflect NFT [15–17]. So far, there is no established fluid biomarker that reflects changes in vasculature. Thus, there is an urgent need to identify and validate new biomarkers that allow monitoring of pathological vascular alterations.

A potential candidate to detect vascular alterations in AD is vascular endothelial growth factor (VEGF). In general, VEGF is essential for the maintenance of the optimal function of the vasculature, but under pathological conditions high levels of VEGF may induce the formation of pathological vessels through angiogenesis [18]. In the CNS, VEGF can be locally produced and secreted by astrocytes and subsequently bind to endothelial VEGF receptors 1 and 2, which in turn activate downstream pathways that regulate cell survival, angiogenesis, and vascular cell permeability [19, 20]. After, for instance, an ischemic stroke or upon CNS injury, VEGF production is induced and may cause cerebral angiogenesis, increased BBB permeability, and dysfunction [21]. Additionally, reduction of the expression of VEGF was reported in cerebral capillaries in postmortem brain tissue derived from patients with AD, indicative of pathological vessel formation [22, 23]. Interestingly, oncological studies have shown that VEGF may serve as a serum biomarker for angiogenic processes that are associated with the progression of different forms of cancer, such as colorectal tumors [24]. In AD, one study showed increased serum VEGF levels in AD patients of microbleeds whereas another study demonstrates [25] lower levels of serum VEGF in patients compared to age-matched controls [26], illustrating its suitability as a potential biomarker for AD.

We hypothesized that VEGF levels in CSF may be altered in AD patients with vascular involvement as evidenced by microbleeds and in VaD patients where vascular pathology is essential to the disease, compared to AD patients without microbleeds and controls.

## Methods

### Patients

We selected patients from the Amsterdam Dementia Cohort [27]. Fifty patients with AD (25 patients with microbleeds and 25 without microbleeds matched for age and gender) and 21 patients with VaD were included and 21 patients with subjective cognitive decline without microbleeds, matched for age and gender, served as the control group. All patients underwent extensive dementia screening at baseline, including physical and neurological examination. Global cognitive functioning was assessed using the Mini-Mental State Examination (MMSE). In addition, our diagnostic workup includes a standardized neuropsychological test battery [27]. Tests included the visual association test (VAT) and the Dutch version of Rey auditory verbal learning task (memory), animal fluency (language), and the trail making test and digit span (attention and executive functions). Diagnoses were made in consensus by a multidisciplinary team. Diagnosis of AD cases was performed following the NIA-AA guidelines [28]. NINDS-AIREN [29] criteria were used to diagnose VaD patients. Individuals who presented with cognitive complaints at our memory clinic but performed normal on clinical investigations (i.e., criteria for MCI, dementia, or any psychiatric disorder not met) served as controls. All subjects gave written consent and the ethical review board of VU Medical Center approved of this study.

### MRI assessment

MRI rating was performed blinded to the patients’ clinical data. MBs were defined as rounded hypointense homogeneous foci up to 10 mm in size in the brain parenchyma on T2*-weighted images. MBs were counted in four lobar regions (frontal, parietal, temporal, and occipital) and in two nonlobar regions: basal ganglia (including thalamus) and infratentorial. To assess the vascular alteration, MRI analysis of cerebral vessels was performed. White matter hyperintensities (WMH) were assessed using the age-related white matter change scale [30]. In addition, the presence of large-vessel and lacunar infarcts was assessed. Large-vessel infarcts were rated as present or absent based on hyperintensity of the lesion on both fluid-attenuated inversion recovery (FLAIR) and T2-weighted sequences. Lacunar infarcts were defined as deep lesions from 3 to 15 mm with low signal on fluid-attenuated inversion recovery and T1 sequences and high signal on T2-weighted images. Lacunar infarcts were scored as present or absent. Furthermore, two widely used visual rating scales for the assessment of atrophy were used. Medial temporal lobe atrophy (MTA) was rated using a 5-point rating scale (0–4) [31]. In the analysis, the average MTA score for the left and right sides was used. Global cortical atrophy was assessed on the fluid-attenuated inversion recovery sequence. The global cortical atrophy scale ranges from 0 to 3. On both scales, maximal atrophy is represented by the highest score.

### Meso Scale Discovery MULTI-SPOT assay system

CSF was collected and stored according to JPND-BIOMARKAPD guidelines [32]. AD CSF biomarkers (Aβ42, total Tau and phospho Tau_181_) were analyzed as a part of the routine diagnosis (Innotest; Fujirebio, Ghent, Belgium) [33].

The VEGF levels in CSF were determined using a Meso Scale Discovery (MSD) cytokine-V-PLEX single cytokine assay (cytokine panel1 human), following the manufacturer’s protocol. The kit was validated for the analysis of VEGF in CSF in earlier studies [34, 35]. Briefly, MSD plates were precoated with capture antibodies on a defined spot. CSF samples were diluted twice using sample dilution buffer and 50 μl of CSF samples were added to each well. The samples were incubated for 2 h while shaking. Plates were washed with 150 μl of washing buffer three times, after which 25 μl of detection antibodies conjugated with electrochemiluminescent labels (MSD SULFO-TAG) were added and were subsequently kept for incubation for 2 h with shaking. Plates were washed again three times using 150 μl of washing buffer and 150 μl reading buffer was added to each well. The MSD buffer added created a chemical environment for electrochemilumiscence. The plates were subsequently analyzed using a MSD imager (Sector Imager 2400) where a high voltage was applied, enabling the captured labels to emit light.

### Statistics

Statistical analysis was performed using SPSS version 20 (IBM,Chicago, IL, USA). The data were checked for normality using the Kolmogorov–Smirnov test. VEGF levels in males versus females and APOEε4 carrier versus noncarriers were analyzed using Student’s *t* test after correcting for age. The four groups were compared using ANCOVA with correction for age and sex. Correlations were performed using Spearman’s correlation test. Statistical significance was defined at (two-tailed) *p* < 0.05.

## Results

### Patient demographics

**Fig. 1:**
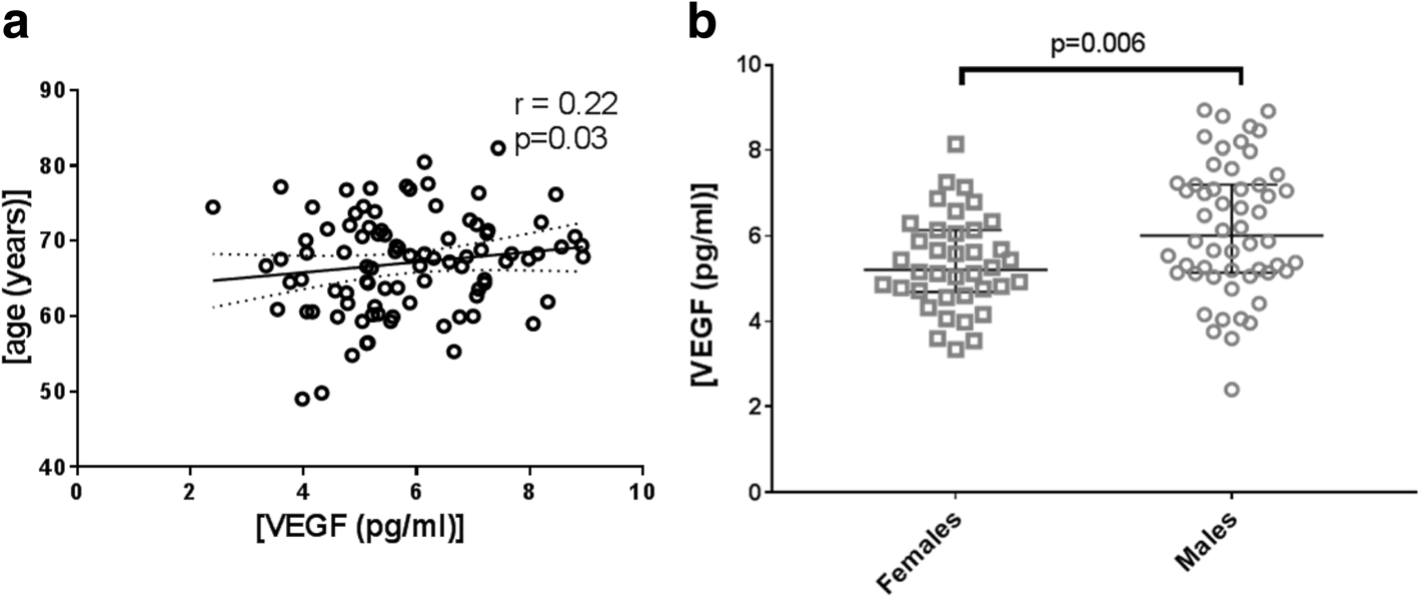
**a** Correlation of CSF VEGF with age. Regression line shown, and dotted lines represent 95% confidence intervals. *p* ≤ 0.05. **b** CSF VEGF levels in males (*n* = 54) and females (*n* = 38). Long horizontal line indicates median, short horizontal line indicates interquartile range. *p* < 0.01. VEGF vascular endothelial growth factor

The demographic and clinical variables are presented in Table 1. The groups differed on Mini Mental State Examination (MMSE) scores and CSF levels of Aβ42, tTau, and pTau (Additional file 1: Figure S1). As per the study design, there was no difference in age and sex between the groups. VEGF levels in the CSF were correlated with age (*r* = 0.22, *p* = 0.03) (Fig. 1a). Additionally, VEGF concentrations were higher in males (*n* = 54) than in females (*n* = 38; *p* = 0.006, adjusted for age) (Fig. 1b).

**Table 1:** Demographic details of patients

	Control	AD–MB	AD+MB	VaD
*N*	21	25	25	21
Sex, female:male	10:11	10:15	11:14	7:15
MMSE^a^	28.5 ± 1.4	21.7 ± 4.3	18.44 ± 5.5	23.50 ± 3.9
Age	65.9 ± 6.1	67.8 ± 6.3	67 ± 7.7	68.6 ± 6.6
Aβ42 (pg/ml)	803 (1057–675)	500 (612–423)	408 (491–304)	606 (868–424)
tTau (pg/ml)	313 (442–240)	615 (743–430)	567 (784–396)	361 (498–196)
pTau (pg/ml)	53 (73–46.50)	85 (109–66.5)	89 (105–68.5)	50 (65–31)
VEGF (pg/ml)	2.7 (3.4–2.3)	2.8 (3.6–2.4)	2.7 (3.2–2.5)	3.1 (3.9–2.6)

MMSE scores and age presented as mean ± standard deviation. Cerebrospinal fluid biomarkers presented as median (interquartile range)

*Aβ42* beta-amyloid, *AD–MD* Alzheimer’s disease without microbleeds, *AD+MB* Alzheimer’s disease with microbleeds, *MMSE* Mini-Mental State Examination, *pTau* phosphorylated tau, *tTau* total tau, *VaD* vascular dementia, *VEGF* vascular endothelial growth factor

^a^A 30-point questionnaire to assess cognitive health

### VEGF levels in the CSF are comparable in controls, AD, and vAD patients and do not correlate with classical AD biomarkers

**Fig. 2:**
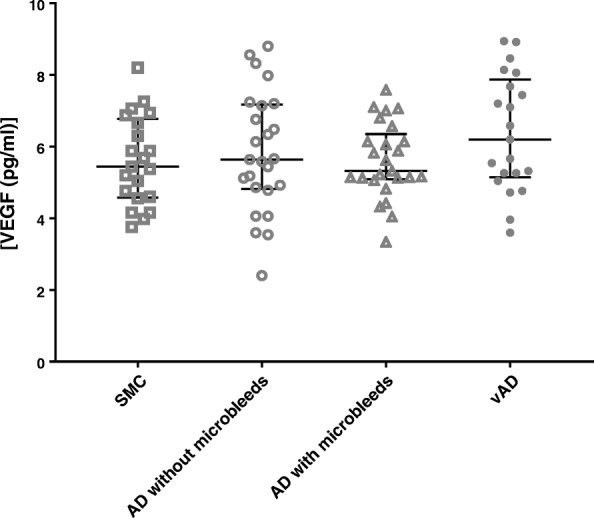
VEGF levels in CSF of patients with subjective memory complaints (SMC) (*n* = 22), Alzheimer’s disease (AD) without microbleeds (AD–MB) (*n* = 25), AD with microbleeds (AD+MB) (*n* = 25), and vascular dementia (VaD) (*n* = 21). Long horizontal line indicates median, short horizontal line indicates interquartile range. VEGF vascular endothelial growth factor

**Fig. 3:**
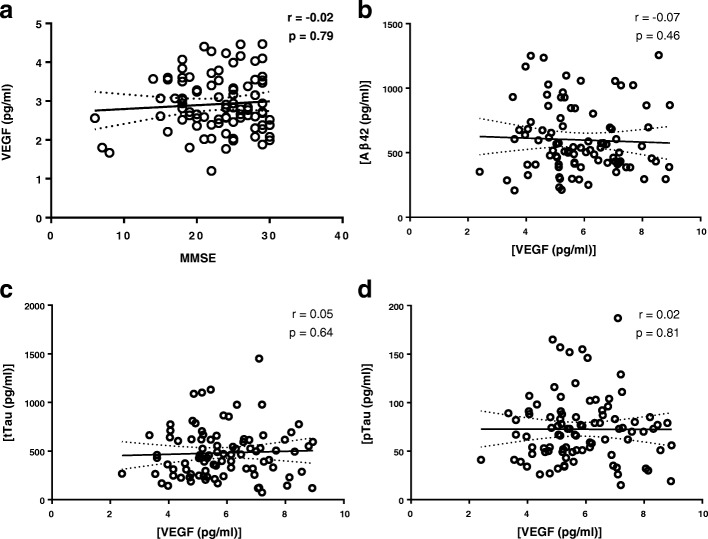
Correlation of CSF VEGF with (**a**) Mini-Mental State Examination (MMSE), (**b**) beta-amyloid (Aβ42), (**c**) total tau (tTau), and (**d**) phosphorylated tau (pTau). Regression line shown, and dotted lines represent 95% confidence intervals. *p* ≤ 0.05. VEGF vascular endothelial growth factor

CSF VEGF levels in controls and AD–MB, AD+MB, and VaD patients were 2.7 ± 1.1, 2.8 ± 1.2, 2.7 ± 0.7, and 3.1 ± 1.3 pg/ml. ANOVA revealed that there were no significant differences in the CSF VEGF levels between diagnostic groups (Fig. 2 and Table 2 and Additional file 2: Table S1). Adjustment for age and sex did not change this result (*F*(3,83) = 0.807, *p* = 0.493). Furthermore, VEGF levels in the CSF did not correlate with MMSE scores (*r* = − 0.02, *p* = 0.79; Fig. 3a), CSF Aβ42 (*r* = − 0.07, *p* = 0.46; Fig. 3b), CSF tTau (*r* = 0.07, *p* = 0.53; Fig. 3c), or CSF pTau (*r* = 0.04, *p* = 0.70; Fig. 3d) levels. Additionally, we found that VEGF levels were similar in APOEε4 carriers and noncarriers (*p* = 0.71).

**Table 2:** Multiple group comparisons of cerebrospinal fluid vascular endothelial growth factor levels^a^

	Sum of squares	df	Mean square	*F*	*p*
Contrast	1.227	3	0.409	0.881	0.455
Error	39.946	86	0.464		

^a^Controls, Alzheimer’s disease without microbleeds, Alzheimer’s disease with microbleeds, and vascular dementia compared using analysis of covariance adjusted for age and sex

## Discussion

The main finding of our study is that VEGF in CSF has similar concentrations across all diagnostic groups, indicating that VEGF in CSF does not help to recognize vascular involvement in patients presenting at a memory clinic. VEGF levels in the CSF were associated with increasing age and with gender, as males had higher levels of VEGF compared to age-matched females.

Our results showed a modest correlation between increasing age and concentrations of VEGF in CSF. Similar to many ageing disorders like atherosclerosis and cardiovascular diseases [36], cerebral vascular distress is also associated with age [37]. The observed association of age and increased levels of VEGF in the CSF may result from an age-related increase of cerebral vascular distress, although a longitudinal study is needed to further understand the relation between VEGF levels in the CSF and increasing age.

We detected that VEGF levels in CSF in males are higher compared to females. A previous study has shown that males have higher concentrations of vascular biomarkers such as E-selectin and vascular cell adhesion molecule-1(VCAM-1) in their CSF [38], which is in accordance with our study. A recent imaging study indicated that cerebrovascular diseases lead to enhanced hippocampal atrophy, independent of Aβ deposition, especially in males [39], suggesting a gender-dependent regulation of vascular function under pathological conditions. Our results may also result from a higher incidence of subclinical cerebrovascular diseases in males than females. This is concordant with an earlier epidemiological study showing that males are at a higher risk of cerebrovascular diseases than females, specifically in those who are middle aged or early old aged [40]. Our finding that CSF VEGF levels are similar across all diagnostic groups is consistent with a previous study in which comparable VEGF levels in the CSF were observed in AD patients (*n* = 23) and controls (*n* = 27) [41]. In general, we did not observe any correlations of VEGF levels with MMSE scores or with the CSF levels of Aβ42, pTau, and tTau, which is in line with previous studies [42]. Our results are also in accordance with data from the ADNI [43], where no differential levels of VEGF were found among control (*n* = 90), mild cognitive impairment (*n* = 130), and AD (*n* = 59) groups at baseline, indicating that VEGF in the CSF may not reflect cerebral vascular distress. Further longitudinal studies are needed to establish whether the correlation between VEGF levels with CSF biomarker Aβ42, pTau, and tTau possibly changes over time during the development of disease. Increased intrathecal VEGF levels in AD (*n* = 17) and VaD (*n* = 19) patients were detected compared to controls (*n* = 18), although this study was limited by the relatively small sample size [44]. Other potential explanations for the differential outcome on the use of VEGF as a biomarker for vascular pathology may be the differences in technologies used and the included sample sizes. Finally, it should be noted that levels of soluble VEGFR1 and VEGFR2, the former of which was shown to be altered in the brain of AD patients [45], modulate angiogenic response of VEGF and, possibly, VEGF levels in CSF.

In this study, for the first time, VEGF levels in CSF were analyzed in four different diagnostic groups including AD patients with and without microbleeds. We used a well-defined cohort where the patients were selected carefully in a specialized memory clinic. Although we had a well-characterized case–control study, the present study was limited by a small sample size. It should be explored further whether VEGF changes longitudinally in AD+MB patients and in VaD patients.

## Conclusion

The present study illustrates that VEGF levels are not altered in the CSF of patients with AD with microbleeds or with VaD. We found higher concentrations, however, in males and with increasing age, suggesting that VEGF may play an important role in cerebral vascular changes related to aging.

## Additional files

Additional file 1: **Figure S1.** Aβ42, tTau, and pTau levels in SMC (*n* = 22), AD (*n* =53), and vAD (*n* = 22) patients. Scatter dot plots show: (left) significant changes in Aβ42 mean concentration between AD vs SMC (*p* < 0.0001), AD vs vAD (*p* = 0.04), and vAD vs SMC (*p* = 0.03); (middle) significant difference in tTau levels between SMC vs AD patients (*p* = 0.0002) and AD vs vAD (*p* = 0.002) but no significant change in vAD and SMC patients; and (c) no significant changes in pTau levels between SMC and vAD patients but significant differences in AD vs SMC (*p* = 0.0004) and AD vs vAD (*p* < 0.0001). Long horizontal line indicates median, short horizontal line indicates interquartile range. (PDF 268 kb)

Additional file 2: **Table S1.** Post-hoc analysis of CSF VEGF levels. (DOCX 15 kb)

## Abbreviations

AD: Alzheimer’s disease
ADNI: Alzheimer’s Disease Neuroimaging Initiative
CSF: Cerebrospinal fluid
MB: Microbleeds
MMSE: Mini-Mental State Examination
VaD: Vascular dementia
VEGF: Vascular endothelial growth factor
