# Supplementary figures and images for: Vascular Endothelial Growth Factor remains unchanged in cerebrospinal fluid of patients with Alzheimer’s disease and vascular dementia
Source: Alzheimers Res Ther. 2018 Jun 23;10:58. doi: 10.1186/s13195-018-0385-8 (PMC6015445; doi:10.1186/s13195-018-0385-8)

Figure S1

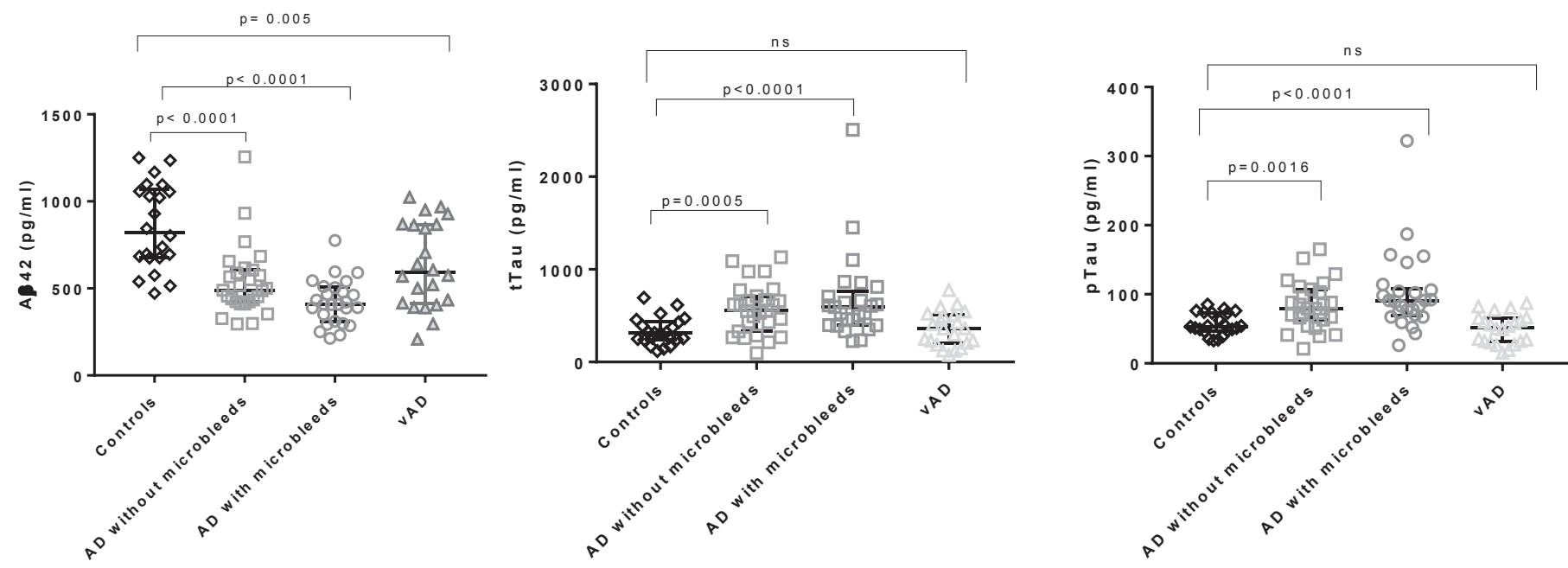

Supplement: Supplementary file 1 — Figure S1. Aβ42, tTau, and pTau levels in SMC (n = 22), AD (n =53), and vAD (n = 22) patients. Scatter dot plots show: (left) significant changes in Aβ42 mean concentration between AD vs SMC (p < 0.0001), AD vs vAD (p = 0.04), and vAD vs SMC (p = 0.03); (middle) significant difference in tTau levels between SMC vs AD patients (p = 0.0002) and AD vs vAD (p = 0.002) but no significant change in vAD and SMC patients; and (c) no significant changes in pTau levels between SMC and vAD patients but significant differences in AD vs SMC (p = 0.0004) and AD vs vAD (p < 0.0001). Long horizontal line indicates median, short horizontal line indicates interquartile range. (PDF 268 kb) [file 13195_2018_385_MOESM1_ESM.pdf]
