# Supplementary material for: Vascular Endothelial Growth Factor remains unchanged in cerebrospinal fluid of patients with Alzheimer’s disease and vascular dementia
Source: Alzheimers Res Ther. 2018 Jun 23;10:58. doi: 10.1186/s13195-018-0385-8 (PMC6015445; doi:10.1186/s13195-018-0385-8)
Supplement: Supplementary file 2 — Table S1. Post-hoc analysis of CSF VEGF levels. (DOCX 15 kb) [file 13195_2018_385_MOESM2_ESM.docx]

**Table S1: Post-hoc analysis CSF VEGF levels^#^**

| (I) Group based on diagnosis and MB status | (J) Group based on diagnosis and MB status | Mean Difference (I-J) | Std. Error | Sig. | 95% Confidence Interval for Difference | |
| --- | --- | --- | --- | --- | --- | --- |
|  |  |  |  |  | Lower Bound | Upper Bound |
| subjective cogntive decline | AD no MBs | -.038 | .203 | .852 | -.442 | .366 |
|  | AD with MBs | .057 | .203 | .778 | -.345 | .460 |
|  | VaD | -.259 | .214 | .231 | -.685 | .168 |
| AD no MBs | subjective cogntive decline | .038 | .203 | .852 | -.366 | .442 |
|  | AD with MBs | .095 | .193 | .622 | -.288 | .479 |
|  | VaD | -.221 | .202 | .279 | -.623 | .182 |
| AD with MBs | subjective cogntive decline | -.057 | .203 | .778 | -.460 | .345 |
|  | AD no MBs | -.095 | .193 | .622 | -.479 | .288 |
|  | VaD | -.316 | .203 | .124 | -.720 | .088 |
| VaD | subjective cogntive decline | .259 | .214 | .231 | -.168 | .685 |
|  | AD no MBs | .221 | .202 | .279 | -.182 | .623 |
|  | AD with MBs | .316 | .203 | .124 | -.088 | .720 |
